# Controlled usage of H/D exchange to circumvent concomitant polymorphs of ROY

**DOI:** 10.1107/S2052252518009995

**Published:** 2018-07-27

**Authors:** Jacqueline Falk, Detlef Hofmann, Klaus Merz

**Affiliations:** a Inorganic Chemistry, Ruhr-University Bochum, Universitaetsstrasse 150, Bochum 44801, Germany; b CRS 4, Piscina Manna 1, Pula 09010, Italy

**Keywords:** concomitant polymorphs, deuteration, hydrogen bonds, ROY, crystal engineering, H/D exchange

## Abstract

The hypothesis that H/D exchange affects the structural formation of organic compounds in the solid state is supported by a deeper understanding of the altering polymorphism of ROY (a substance striking for its high number of polymorphic forms) through deuteration. Therefore, ROY was deuterated at its amine function, which leads to a seemingly small yet effective modification of the hydrogen-bond strength. In contrast to the crystallization of the non-deuterated ROY in methanol or ethanol, which leads to the simultaneous formation of two forms (OP and Y polymorphs), so-called concomitant polymorphs, the crystallization of *d*_1_-ROY leads to the selective formation of the Y polymorph exclusively. The preferred aggregation behavior of the Y form of *d*_1_-ROY is assigned to the weakening of an intramolecular hydrogen bond and a consequently strengthened intermolecular hydrogen bond after deuteration.

## Abstract 2

Deuteration of ROY at its amine function allowed the circumvention of concomitant polymorphs and produced exclusively the yellow Y polymorph of *d*_1_-ROY.

## 1. Introduction   

Recently, deutetrabenazine, the first deuterated drug, has been approved by the FDA (Mullard, 2017 ▸). This is one reason for the remarkable upcoming interest in deuterated organic compounds during recent years. In the case of deutetrabenazine, the H/D exchange slows down the rate of drug metabolism. Such kinetic isotope effects can be explained by the lower zero-point energy of deuterium, which implies a smaller vibrational amplitude and hence a smaller effective radius than that of protium (Ubbelohde & Gallagher, 1955 ▸). However, the impact of deuterium substituents is not limited to reducing metabolism. A detailed survey of deuterated and nondeuterated compounds indicates that isotopic substitution can influence the molecular arrangement in the solid state (Merz & Kupka, 2015 ▸). The phenomena of structural changes in crystals induced by isotopic substitution has been proposed as ‘isotopic polymorphism’. Furthermore, recent studies have shown that heavy water allows the control of reaction pathways by obtaining an alternative crystalline reaction product (Enkelmann *et al.*, 2017 ▸).

In contrast to covalent bonds, which show electron pairing, hydrogen bonds show group properties. Their energies and geometries are functions of the hydrogen-bonded pattern in the solid state. Molecular surroundings, solvation and ionic radii influence those patterns and therefore the whole hydrogen-bonded network in the crystal lattice. The H/D isotope effect leads to alterations in the geometry of hydrogen bonds with anharmonic potential.

The impact of modified hydrogen bonds on the aggregation behavior of molecules in the solid state, which is observable for several compounds (Merz & Kupka, 2015 ▸), could be a suitable approach to avoid the simultaneous crystallization of concomitant polymorphs under the same crystallization conditions. The controlled formation of polymorphs is crucial in chemical manufacture, especially in the pharmaceutical industry where consistency and reliability are of importance (Jiang *et al.*, 2008 ▸). Concomitant polymorphism is the result of an interplay between thermodynamics and kinetics. The lattice-energy differences of the obtained polymorphic forms are typically very small; over half of the polymorph pairs are separated by less than 2 kJ mol^−1^, and lattice-energy differences exceed 7.2 kJ mol^−1^ in only 5% of cases (Nyman & Day, 2015 ▸).

Focusing on controlled polymorphic behavior of organic substances, this study demonstrates the usage of H/D exchange to circumvent concomitant polymorphs of the model compound ROY {5-methyl-2-[(2-nitrophenyl)­amino]-3-thiophenecarbonitrile}. ROY is famously named for its numerous colorful [red (R), orange (ON, OP) and yellow (Y, YN)] crystalline modifications, with ten explored polymorphs to date (Yu, 1995 ▸) from which seven have been structurally characterized (Yu *et al.*, 2000 ▸; Yu, 1995 ▸, 2002 ▸).
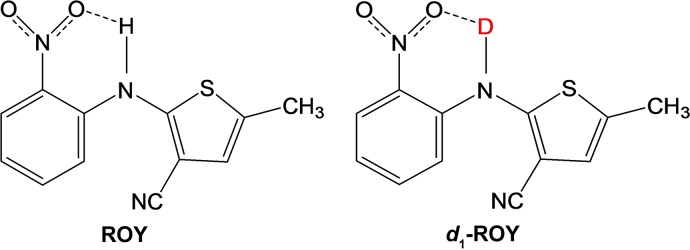


The crystal packing of a variety of different polymorphs of ROY is dominated by π–π stacking of the nitrophenyl and thiophenyl moieties. In this work, we focus on the crystallization of ROY and *d*_1_-ROY with a deuterated amino group from ethanol and methanol. It is well known that different polymorphs can be obtained based on the solvents used. The crystallization of ROY in solvent showed poor polymorphic selectivity, often several polymorphs nucleate simultaneously. There are hints in the literature that six polymorphs can be obtained from crystallization in methanol (Yu *et al.*, 2000 ▸). The same source also explains ‘rough guidelines’ for the syntheses of the polymorphs Y, ON, R, OP and YN; however, this source does not provide details about the solvent type. More specific are the investigations by Lee (2014 ▸), which state the influences of concentration and solvent type on the formation of ROY polymorphs. Crystallization of ROY in methanol leads to the formation of the concomitant polymorphs Y and OP. The great variety of different polymorphic forms of ROY means their relative stability is an interesting aspect. A free-energy–temperature diagram of ROY polymorphs constructed from melting and eutectic melting data was published by Yu and coworkers (Yu, 2010 ▸). Between 40 and 70°C, the relative stability follows the order of Y (most stable) > ON ≃ OP > YT04 > R >YN > ORP. The differences in the free energy of the polymorphs are rather small and the crystal energies show only minor discrepancies of ∼2 kJ mol^−1^ (Yu, 2010 ▸) and especially in the cases of Y and OP polymorphs which are less than 0.4 kJ mol^−1^. If polymorphic conversion is slow, polymorphs can coexist under the same conditions at the same time, which means they can form concomitant polymorphs.

## 2. Polymorphic screening of ROY   

**Figure 1:**
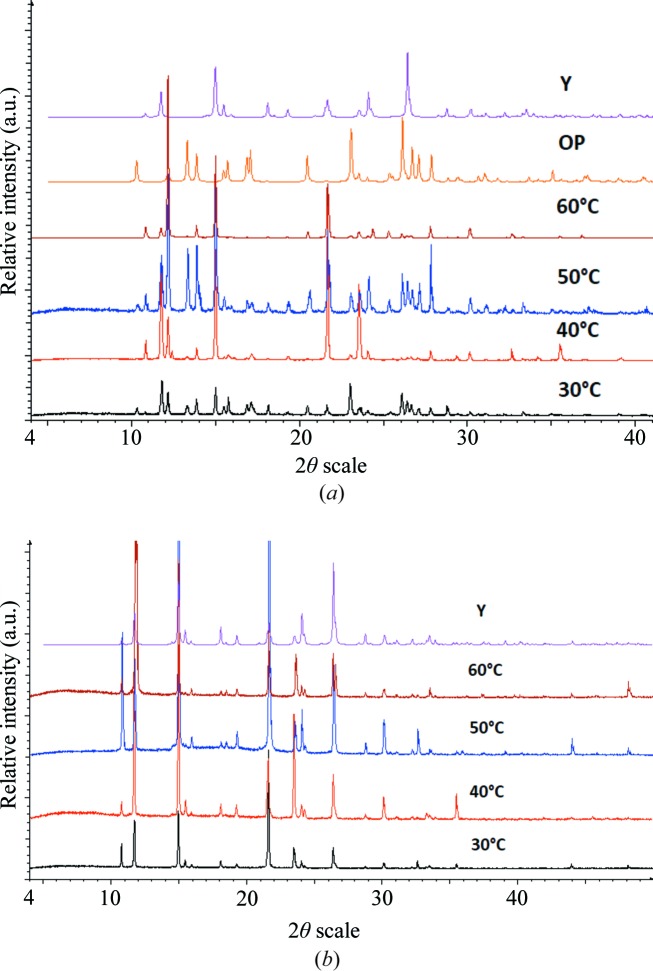
Powder diffractograms of the temperature screening of (*a*) ROY and (*b*) *d*_1_-ROY from 30 to 60°C (with slow cooling) and the reference diffractograms of OP and Y.

The focus of the investigation was to gain a deeper understanding of the crystallization behavior of ROY and compare it with that of *d*_1_-ROY. First, screening involved typical solvent crystallization experiments. Apart from external factors such as temperature or pressure, the solvent is the central influencing factor on the crystallization pathway. In these experiments, a specific amount of ROY was dissolved at 50°C in one of the selected solvents (ethyl acetate, di­chloro­methane, methanol and ethanol) and was slowly cooled down to −7°C. The concomitant polymorphs, which could be obtained *via* solution crystallizations, are always in the Y and OP forms. The obtained products were analyzed by powder X-ray diffraction (PXRD). During the solvent crystallizations, we found that the formation of ROY polymorphs is temperature dependent. As a consequence, a temperature screening was performed in order to gain a deeper understanding of the existence of concomitant polymorphs. The diffractograms of the obtained crystals from 30 to 60°C are shown in Fig. 1 ▸(*a*) and are compared with the reference diffractograms of forms Y and OP. The temperature screening was performed with a saturated solution of ROY in methanol at a given temperature and was then slowly cooled to −7°C. All experiments in methanol resulted in the concomitant polymorphs Y and OP. However, the ratio of Y:OP changes; at higher temperatures the percentage of Y form decreases. The temperature screening was also performed with rapid cooling of the solution after bringing it to the specified temperature. In these experiments, kinetic factors played a major role, which resulted in an unpredictable mixture of concomitant polymorphs. The experiments at 30 and 40°C show the same results as those involving slow cooling. Both concomitant polymorphs Y and OP were obtained. Above 45°C, another form is present in the mixture that can be identified as YN (see Fig. S1 of the supporting information). The formation of concomitant polymorphs Y and OP was also observed during the crash-cooling crystallization of solid ROY. In this experiment, a specified amount of ROY was heated above 120°C until it was completely melted and was then rapidly cooled down with liquid nitro­gen to approximately −190°C. The obtained orange and yellow crystalline solids were identified using PXRD as the Y and OP forms.

## 3. Synthesis and polymorphic screening of *d*_1_-ROY   

An analysis of the polymorphic character of *d*_1_-ROY was conducted in order to highlight the differences between ROY and *d*_1_-ROY. The aim was to deuterate ROY at its amine functional group. ROY was deuterated by heating at 70°C for 20 min in a saturated solution of *d*_6_-ethanol or *d*_4_-methanol. The solutions were cooled to −7°C. After the deuteration of ROY, yellow crystals were obtained and then analyzed by IR spectroscopy, single-crystal X-ray diffraction and PXRD (Table S1).

Following the approach of polymorphic screening of ROY, a specific amount of *d*_1_-ROY was dissolved at 50°C in one of the selected solvents (*d*_8_-ethyl acetate, *d*_2_-dichloromethane, *d*_4_-methanol and *d*_6_-ethanol) and was slowly cooled to −7°C. The only polymorph obtained in all of these crystallization experiments was the polymorphic form Y. Furthermore, the temperature-screening crystallization experiments of a saturated solution of *d*_1_-ROY in *d*_4_-methanol from 30 to 60°C with slow cooling to −7°C produced only the Y form of *d*_1_-ROY (Fig. 1 ▸ *b*). The temperature screening was also performed with fast cooling of the solution after bringing it to the specified temperature. The patterns measured by PXRD are comparable with the reference of form Y.

**Figure 2:**
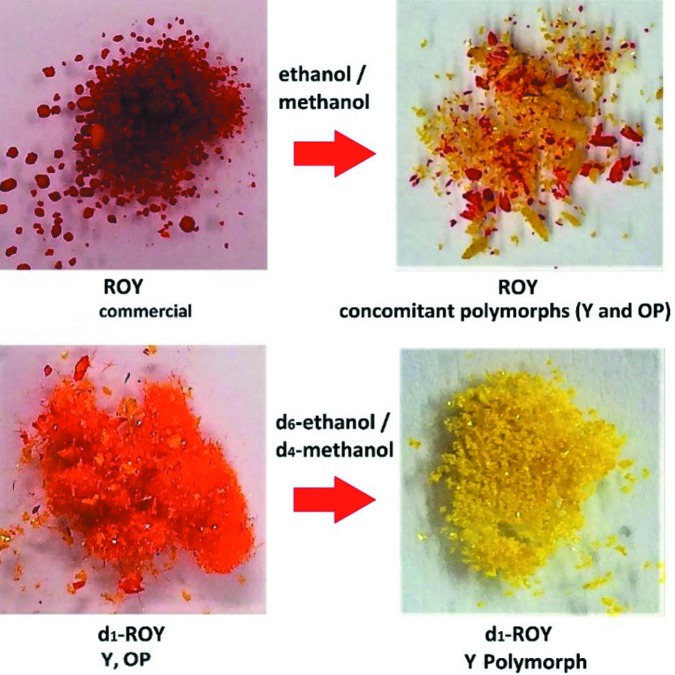
Crystallization of ROY yields concomitant polymorphs (top); crystallization of *d*_1_-ROY under the same conditions gives the Y polymorph.

The crystallization experiments performed show that crystallization of ROY in either ethanol or methanol yields concomitant yellow and orange Y and OP polymorphs, whereas the crystallization of *d*_1_-ROY under the same conditions yields exclusively the yellow form Y, as illustrated in Fig. 2 ▸.

The H/D exchange reaction of ROY and *d*_1_-ROY is also reversible. It is possible to dissolve *d*_1_-ROY in methanol and heat it at 60–70°C for 20 min to obtain the concomitant form Y and OP of non-deuterated ROY. Therefore, the experiments lead to the definition of the scheme below, which highlights the selectivity of the deuterated Y polymorph under 60°C and the reversible deuteration of ROY.
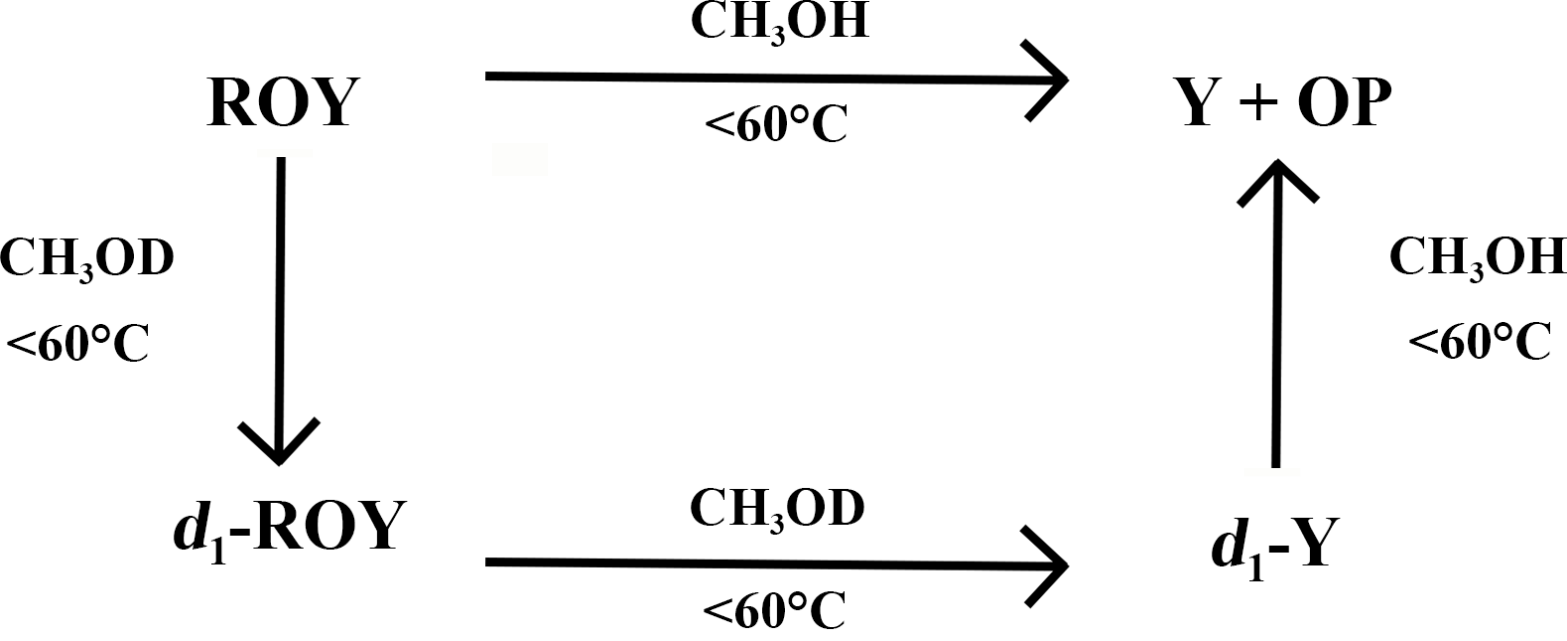


## 4. Interpretation of the aggregation of the *d*_1_-Y polymorph in the solid state and energetic aspects   

The crystallographic data of the Y polymorph of ROY and *d*_1_-ROY show only small differences (Table S1). The cell volume of *d*_1_-ROY is slightly larger than that of ROY. Table 1 ▸ compares the intermolecular and intramolecular bond lengths of the Y polymorph in ROY and *d*_1_-ROY.

**Figure 3:**
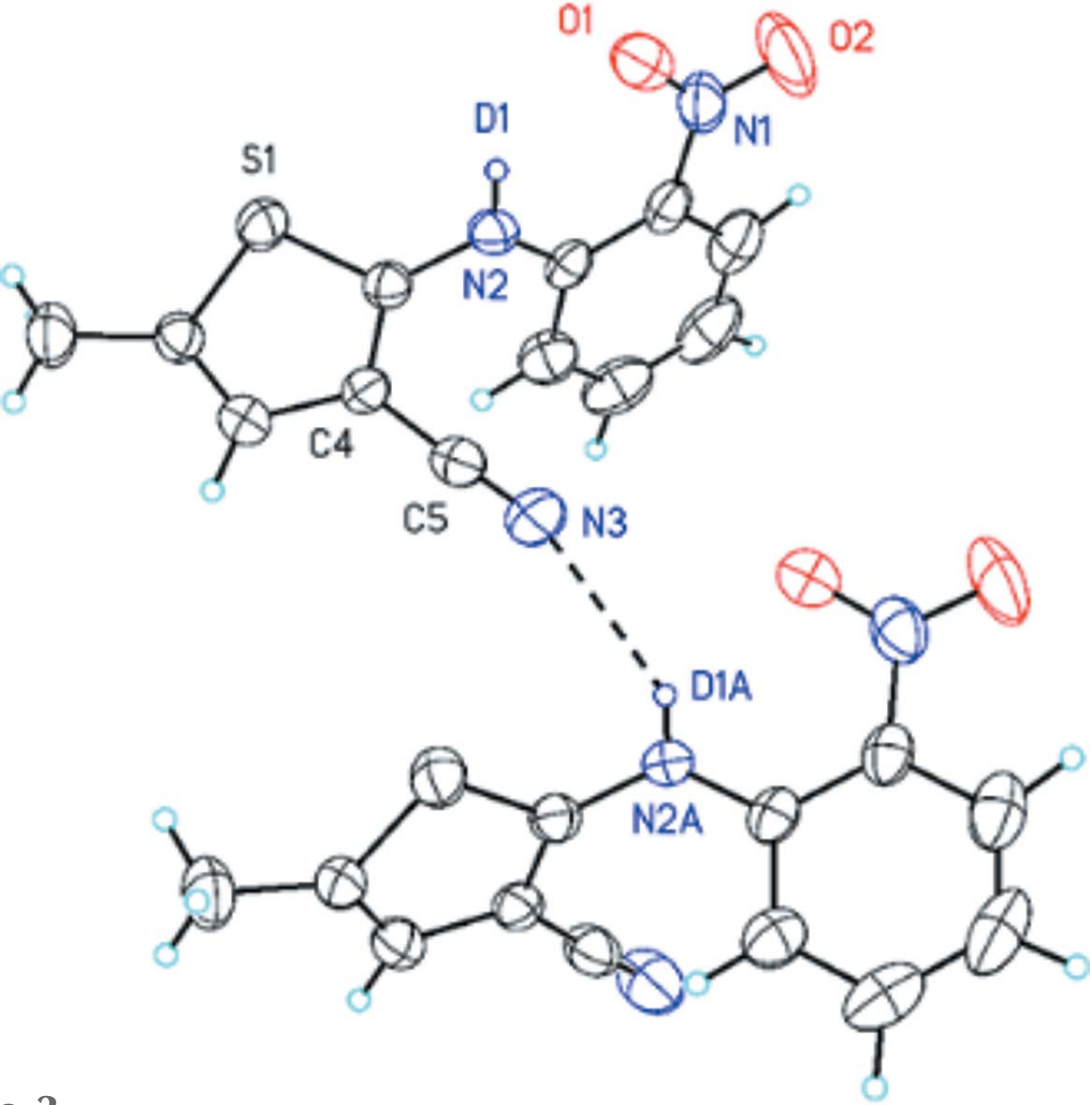
Molecular aggregation of the Y form of d_1_-ROY and the intermolecular hydrogen bond N—D⋯NC (2.288 Å).

Compared with the other polymorphic forms of ROY, the crystal packing of the deuterated and non-deuterated Y polymorph is dominated more by π⋯π stacking interactions of the neighboring nitrophenyl groups and neighboring thiophene and nitrophenyl moieties. The distances between the centers in the crystal structures of ROY and *d*_1_-ROY differ slightly. In contrast to the other polymorphic forms of ROY, the crystal packing of the Y form features an additional intermolecular hydrogen bond (N—H/D⋯NC). However, this seemingly unimpressive and weak intermolecular hydrogen bond provides an opportunity to influence the energy parameter through H/D exchange, resulting in the thermodynamically preferred crystalline polymorph. As a matter of fact, the H/D exchange leads to a reduction of the intermolecular hydrogen-bond distance *D*⋯*A* from 2.420 Å (N—H⋯NC) in ROY to 2.288 Å (N—D⋯NC) in *d*_1_-ROY (Fig. 3) ▸.

The H/D substitution results in the preferred formation of polymorph Y, with the aggregation illustrated in Fig. 3 ▸. From this, we can conclude that the intermolecular interactions in this aggregation become favorable upon substitution. The shortest and strongest interaction is the H/D⋯N bond and we studied this interaction in detail. To understand the effects of H/D exchange, data mining studies of known deuterium compounds and their undeuterated analogs were undertaken.

The strength of the intermolecular pair interactions can be calculated by data mining on experimental structures (Kuleshova & Hofmann, 2010 ▸). To derive the potentials, we made use of the fact that any experimental structure is a local minimum in the Gibbs energy. This allowed us to create two classes of structures: the first contains all of the experimental structures, which must have negative Gibbs energies, otherwise the structures would be unstable; the second class contains distorted structures minus the experimental structure. Since the experimental structure is a local minimum, all elements of the scond class must have a positive Gibbs energy. During data mining, the parameters of the force field are trained to assign all elements to the right class as effectively as possible. As a result, the overall procedure optimized the parameters *a_i_* of any model (force field) in order to assign the experimental structures to a local minimum. The function that fulfills this condition is, by definition, the Gibbs energy. In any given case, the Gibbs energy is approached by atom pair potentials *g*, developed as a Taylor series of the inverse cubes of distances *r*: 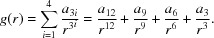



Recently, the algorithm behind the derivation of the data-mining force fields has been published (Hofmann & Kuleshova, 2018 ▸).

**Figure 4:**
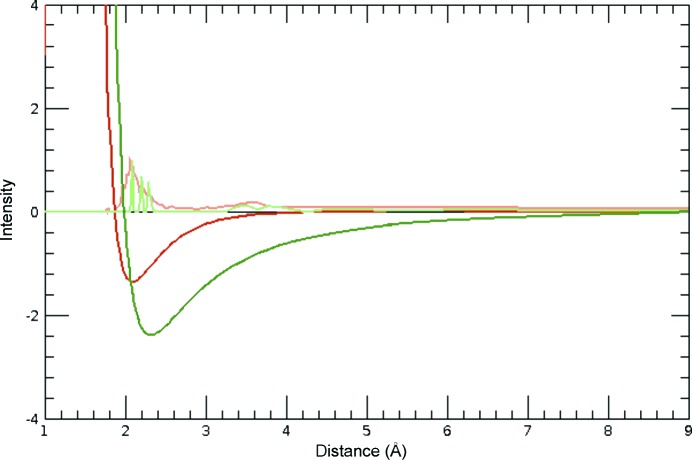
Radial distribution functions (light green and light red) and calculated effective intermolecular potentials of N—H/NC (red) and N—D/NC (green) interactions.

The result in Fig. 4 ▸ shows that the N⋯H potential is affected by substitution with deuterium; it is lowered and shifted to shorter distance. A comparable phenomena is observed in acridine and deuterated acridine (Kupka *et al.*, 2012 ▸). The stabilization of the C—H⋯N intermolecular interaction by H/D-substitution leads, in the case of deuterated acridine, to the polymorphic form III and prevents the rearrangement into the more stable form II.

In the case of concomitant polymorphism of the ROY polymorphs Y and OP, which possess a similar free energy, it is possible to favor crystallization of one polymorph through the deuteration of the amino function, which includes an additional weak intermolecular hydrogen bond. The presented investigations illustrate the far-reaching possibilities of H/D exchange, which can be applied as a ‘precision-engineering tool’ in crystallization processes, where weak intermolecular interactions can become the major controlling influence in the competition of concomitant polymorphs.

## Supplementary Material

Supporting Information. DOI: 10.1107/S2052252518009995/ed5015sup1.pdf

## Figures and Tables

**Table 1:** Intermolecular and intramolecular bond lengths of the Y polymorph in ROY and *d*_1_-ROY

	ROY distance (Å)	*d*_1_-ROY distance (Å)
π⋯π (nitro­phenyl⋯nitro­phenyl)	3.375 (center⋯center)	3.451 (center⋯center)
π⋯π (nitro­phenyl⋯thio­phene)	4.904 (center⋯center)	5.050 (center⋯center)
Intermolecular bond (*D*⋯*A*), N—H/D⋯NC	2.420 (N⋯N)	2.288 (N⋯N)
Intramolecular bond (H/D⋯*A*), N—H/D⋯ON	1.997 (H⋯O)	2.098 (D⋯O)
