# Supplementary material for: Controlled usage of H/D exchange to circumvent concomitant polymorphs of ROY
Source: IUCrJ. 2018 Jul 27;5(Pt 5):569–73. doi: 10.1107/S2052252518009995 (PMC6126644; doi:10.1107/S2052252518009995)

IUCrJ

Volume 5 (2018)

Supporting information for article:

Controlled usage of H/D-exchange to circumvent concomitant polymorphs of ROY

Jacqueline Falk, Detlef Hofmann and Klaus Merz

S1. Synthesis of d_1-ROY

d_1-ROY was synthesized *via* a deuterated solvent reflux reaction. A saturated solution at RT of ROY in d_4-methanol, d_6-ethanol or d_8-1-propanol was stirred and heated under reflux for 30 minutes. The solution was slowly cooled down to -7 °C. The product was obtained as a yellow crystalline product. IR (υ [cm^{-1}]): 2442, 2230, 1613, 1568, 1552, 1364, 1228, 1263, 930, 839.

S2. Temperature Screening

ROY and d_1-ROY were dissolved until saturation in d_6-methanol at temperatures between RT and 60 °C and subsequently the solution was slowly cooled down to -7 °C. For comparative reasons, a temperature screening of ROY was carried out similarly. To reproduce the results of the crystallization investigations, the experiments were repeated. The obtained crystalline materials were characterized by powder X-Ray diffraction (PXRD), measured on a Bruker AXS Advance diffractometer in flat mode and Bragg-Brentano geometry using filtered CuKα radiation.

Table S1. Crystallographic data of the Y polymorph of d_1-ROY and ROY.

	d_1-ROY Y polymorph	ROY Y polymorph (Yu *et al.*, 2000)
identification code	CCDC 1828199	CCDC 1241884
empirical formula	$C_{12}H_8D_1N_3O_2S_1$	$C_{12}H_9N_3O_2S_1$
formula weight	260.28	259,28
Temperature	170	273
crystalline system	monoclinic	monoclinic
space group	$P2_1/n$	$P2_1/n$
description	yellow prism	yellow prism
a [Å]	8.537(5)	8.5001(1)
b [Å]	16.466(5)	16.413(2)
c [Å]	8.569(5)	8.5371(1)
β [°]	91.905(5)	91.767(7)
V [$Å^3$]	1203.9(11)	1190.5(4)
Z	4	4
D_{calcd}, [g/cm^3]	1.407	1.447
F(000)	536	536
2Θmax	50	48
reflections measured	14600	2095
Unique reflections	2837	1956
parameters	199	167
R1 with $I>2\sigma(I)$	0.0400	0.033
wR2 for all reflections	0.0829	0.044
GOF	0.761	1.415

Figure S1. Temperature crystallization screening of ROY from methanol at different temperatures and fast cooling down to -7°C

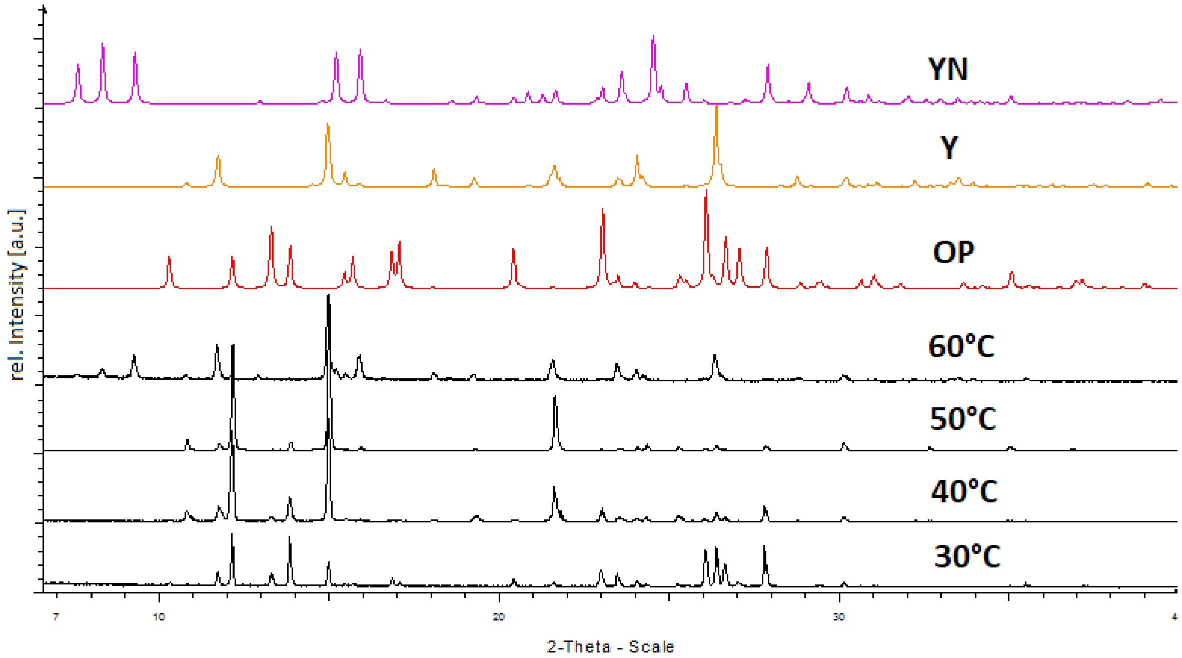

Supplement: Supplementary file 1 [file m-05-00569-sup1.pdf]
